# Factors associated with complexity during transvenous lead extraction and confirmation of a very-low-risk subset

**DOI:** 10.1016/j.hroo.2025.11.004

**Published:** 2025-11-11

**Authors:** Emma Francis, John A. Anderson, Emmanuel Danso, William Devries, Tanner Robl, Nicholas Kettelkamp, Jacob Cushing, Loren D. Berenbom, Rhea Pimentel, Y. Madhu Reddy, Amit Noheria, Seth H. Sheldon

**Affiliations:** Department of Cardiovascular Medicine, The University of Kansas Health System, Kansas City, Kansas

**Keywords:** Lead extraction, Pacemaker, Implantable cardioverter-defibrillator, Laser extraction, Mechanical extraction, Snare

## Abstract

**Background:**

Transvenous lead extraction/explantation (TLE) requires significant resources/coordination.

**Objective:**

We sought to assess factors associated with the need for complex tools or a major intraoperative complication (complexity) with TLE.

**Methods:**

This was a single-center, retrospective cohort study in patients undergoing TLE between January 2010 and March 2022.

**Results:**

There were 930 TLEs in 868 patients (age 62.9 ± 15.8 years, 68% men, 63% implantable cardioverter-defibrillators [ICDs], 1588 extracted leads, 29% infected). The median age of the oldest lead was 4.9 years (interquartile range, 1.9–8.1). Complex tools were used for 59.9% of TLE, including the laser (37.2%), laser and mechanical sheaths (9.2%), mechanical sheath (9.1%), snare and laser/mechanical (4.2%), and snare alone (0.1%). Complete success was achieved in 892 TLEs (95.9%). Complexity occurred with 59.9% of TLE (n = 557). Unplanned complexity occurred in only 2 of 203 pacemakers (1.0%), with the oldest lead being <2 years old and ICDs with the oldest lead being <1 year old. Factors independently associated with complexity included: number of ICD leads (*P* < .0001), combined lead age (*P* < .0001), number of transvenous leads (*P* < .001), abandoned leads (*P* = .0058), age at the time of implantation (*P* = .019), oldest lead age (*P* = .025), and patient age at TLE (*P* = .025). Major intraoperative complications occurred with 10 TLEs (1.1%). No major intraoperative complications occurred with traction alone.

**Conclusion:**

Factors associated with complexity included the presence of an ICD, abandoned leads, patient age, and the number/age of leads. The risk of unplanned complexity is low for pacemaker leads <2 years and ICDs <1 year since implantation. This subset can be considered for TLE in the electrophysiology laboratory without immediate cardiac surgical backup.

## Graphical abstract

**Figure:**
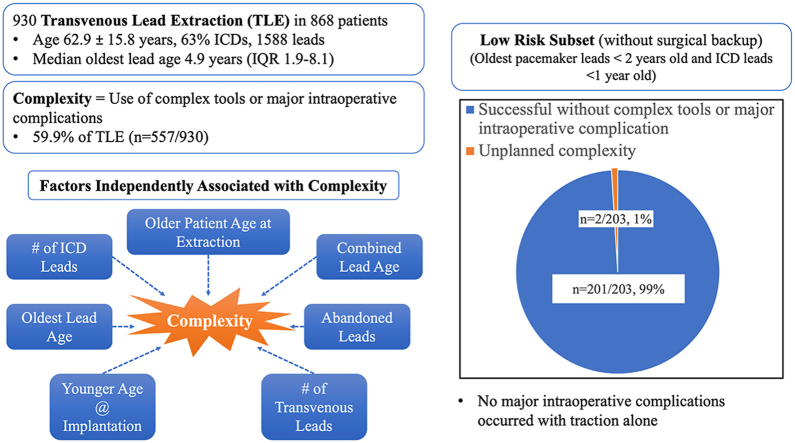

**Key Findings**

▪ The number of implantable cardioverter-defibrillator (ICD) leads, combined lead age, number of leads, presence of abandoned leads, patient age at implantation, patient age at transvenous lead extraction (TLE), and time since implantation of the oldest lead were independent predictors of complexity with TLE.
▪ There was a low frequency of major intraoperative complications (1.1%) with TLE.
▪ There was a very low likelihood of needing complex tools or a major intraoperative complication for recently placed leads (<2 years for pacemaker and <1 year for ICD leads).

## Introduction

Transvenous lead extraction/explantation (TLE) is occasionally necessary if a cardiac implantable electronic device (CIED) is infected or has a lead fracture/failure or to facilitate device upgrade. The reported clinical success of TLE is high (97.7%), and the frequency of major adverse events is low at 1.4%.^1^ Many TLEs are performed in hybrid operating rooms (and occasionally in electrophysiology [EP] laboratories) with cardiac surgical availability. There may be a subset of patients with a low risk of needing complex tools for successful TLE and a low risk of major adverse events. The 2017 Heart Rhythm Society (HRS) expert consensus statement on CIED lead management and extraction recommends that a “cardiac surgeon and surgical team are immediately available with access to equipment to perform emergent sternotomy or thoracotomy within 5 to 10 minutes.”^2^

Given that TLE programs require significant resources and coordination, many centers attempt TLE without complex tools in a very-low-risk patient subset in the EP laboratory without cardiac surgical backup. At our center, we consider this approach in select patients with pacemakers with the oldest lead being <2 years old and implantable cardioverter-defibrillators (ICDs) with the oldest lead being <1 year old. A better understanding is needed of the factors associated with the risk of either needing complex tools or a major intraoperative complication.

We sought to assess the frequency of and factors associated with the need for complex tools or a major intraoperative complication (complexity) at the time of TLE. We hypothesized that patients with recent CIEDs undergoing TLE (pacemakers with the oldest leads implanted within 2 years or ICDs within 1 year) have a low likelihood of unplanned complexity.

## Methods

This single-center, retrospective study included patients aged ≥18 years who underwent attempted lead extraction or lead explantation between January 2010 and March 2022 at a large-volume academic center. There were 9 experienced EP extractors. Lead extraction was defined as the removal of leads when they required the assistance of equipment not typically required during implantation or when at least 1 lead was implanted for >1 year.^2^ A lead explant procedure was defined as all leads being removed without tools or with implantation stylets and when all leads removed were implanted for <1 year. Patients with only leadless pacemakers or subcutaneous ICDs were excluded. The study was approved by the Institutional Review Board on Human Research at The University of Kansas Health System (the need for consent was waived) and was conducted in accordance with the Declaration of Helsinki.

Most TLEs were performed in the hybrid operating room with general anesthesia with cardiac surgery available. The surgeon was in close proximity during the critical aspects of the TLE and was often in the operating room. The cardiac perfusion team was in the room during the TLE. A subset of patients in whom the oldest pacemaker leads were <2 years old or with an ICD system with the oldest lead being <1 year old underwent TLE in the EP laboratory with moderate sedation or monitored anesthesia care without immediate cardiac surgical backup. In these instances, a cardiac surgical team was present at the hospital if needed.

Tool selection and approach were at the discretion of the TLE operator. Most operators initially used a superior approach using traction, a laser sheath, or a mechanical tool if necessary. If the leads had been previously cut without adequate availability of a “rail” for a superior approach, a femoral approach was used. If the procedure included placement of new leads, a venogram or new access was often obtained before TLE. If the venous system was occluded, attempts were made to preserve access on the ipsilateral side. Lead preparation included attempting to retract the helix for active fixation leads, utilization of a locking stylet, suturing the insulation with half-hitches, and securing the conductor cables (for ICD leads). Locking stylets used in the study included the Philips lead locking device (various sizes) and the Cook Liberator. If traction with the locking stylet was unsuccessful, the operator would often then use a sheath (Cook Byrd dilator), laser (Philips Glide Light 12F/14F/16F), or mechanical sheath (Philips TightRail 11F/13F, TightRail Sub-C, Cook Evolution, or Evolution Shortie RL).

Complete procedural success was defined as a TLE procedure with removal of all targeted leads and all material from the vascular space with the absence of any permanently disabling complication or procedure-related death.^2^ Clinical success was defined as removal of all targeted leads and lead material from the vascular space or retention of a small portion of the lead (<4 cm) that does not negatively affect the outcome goals of the procedure. Partial success was defined as the removal of some but not all intended leads. Failure was defined as attempted TLE where complete procedural or clinical success cannot be achieved or the development of any permanently disabling complication or procedure-related death.

Major and minor complications were defined according to the 2017 HRS expert consensus document.^2^ Major complications included death, cardiac avulsion, vascular laceration, respiratory arrest, cerebrovascular accident, pericardial effusion requiring intervention, hemothorax requiring intervention, cardiac arrest, thromboembolism requiring intervention, flail tricuspid valve leaflet requiring intervention, or massive pulmonary embolism. Minor complications included pericardial effusion without intervention, hematoma requiring evacuation, venous thrombosis requiring medical intervention, vascular repair at venous entry site, migrated lead fragment without sequelae, bleeding requiring transfusion, arteriovenous fistula requiring intervention, coronary sinus dissection, pneumothorax requiring chest tube, worsening tricuspid valve function, or pulmonary embolism. A major intraoperative complication was defined as a complication requiring emergent pericardiocentesis, cardiac surgical intervention, or death.

The indications for TLE were classified as lead malfunction, infection, access/planned upgrade, or other. Patient characteristics, device types, and lead details were obtained from the medical record. Combined lead age was defined as the sum of the lead dwelling times (time between implantation and TLE). The lead removal intent was categorized as a full system TLE (TLE of all transvenous leads) or partial system TLE (removal of some but not all leads).

The use of complex tools was defined as the use of equipment not typically required during implantation. Complexity was defined as the use of complex tools (assistance of equipment not typically required during implantation with the exception of lead locking devices/locking stylets) or a major intraoperative complication. Complexity was specified further as unplanned or planned complexity. Unplanned complexity was defined as complexity encountered during the procedure that was either not planned for prior to the procedure or a major intraoperative complication. In anticipation of complexity, patients with pacemakers in whom the oldest pacemaker leads were >2 years old or with ICD systems with the oldest lead being >1 year old underwent TLE in the hybrid room with cardiac surgical backup. Some patients with <2-year-old pacemaker leads or ICD systems with the oldest lead being <1 year old had planned complexity, such as known venous occlusion (extracting for access) or lead masses/vegetation with planned complex tool utilization.

### Statistical analysis

Categorial and continuous variables were presented as frequency/percentages and mean/median, respectively. For continuous variables with skewed distribution, median values with interquartile range (IQR) were reported. Outcomes were compared between groups using the Pearson χ^2^ test or Fisher’s exact test as deemed appropriate for categorical variables and the 2-sample Student *t* test or Wilcoxon rank sum test for continuous variables as deemed appropriate. JMP Pro version 17.2.0 (SAS, Cary, NC) was used for statistical testing, and 2-sided *P* values of < .05 were statistically significant. Logistic regression analysis was used to determine the independent predictors of complexity. Logistic regression was done in a stepwise manner (removing 1 nonsignificant parameter at a time) using characteristics with univariate *P* values of <.20 as candidate variables.

## Results

### Patient demographics

There were 930 TLEs among 868 patients (Table 1) (age 62.9 ± 15.8 years, 68% men, 63% ICDs, 28% cardiac resynchronization therapy devices, 2091 total endocardial leads, 1588 leads extracted, 35 leads with abandoned attempts at TLE). The median time since implantation of the oldest lead was 4.9 years (Table 2) (IQR, 1.9–8.1), and the combined lead age was 9.5 years (IQR, 3.6–16.9). There were 77 lead explant procedures (8.3%) in devices with the oldest lead being <1 year old. There were 2.25 ± 0.76 transvenous leads present per patient. The predominant indication for TLE was noninfectious (n = 659; 71%). Among the 576 TLEs with an ICD lead (66%), 458 (80%) had a dual-coil ICD lead. Noncoronary sinus passive fixation leads were present in 171 TLEs (18.4%). Only 3 TLEs included conduction system pacing leads (0.3%). There were 167 TLEs (18.0%) with a combined lead age of >20 years and 172 TLEs (18.5%) with the oldest lead being >10 years old.

**Table 1:** Patient baseline characteristics

Patient characteristics	Overall (n = 930)
Age (y)	62.9 ± 15.8
Age at oldest lead implant (y)	57.0 ± 17.1
Men	636 (68)
Body mass index (kg/m^2^)	30.5 ± 7.0
Left ventricular ejection fraction (%)	41.6 ± 16.1
Hypertension	591 (64)
Diabetes mellitus	292 (31)
Coronary artery disease (previous PCI/CABG)	447 (48)
Previous sternotomy	264 (28)
Previous stroke	90 (10)
Chronic kidney disease	223 (24)
End-stage renal disease on hemodialysis	30 (3)
Atrial fibrillation	365 (39)
Previous VT/VF or cardiac arrest	283 (30)

CABG = coronary artery bypass graft; PCI = percutaneous coronary intervention; VF = ventricular fibrillation; VT = ventricular tachycardia.

**Table 2:** Patient device characteristics

Device details and extraction indications	Overall (n = 930)
Device types	
Pacemaker	342 (37)
ICD	588 (63)
CRT	257 (28)
Left-sided device only	819 (88.1)
Right-sided device only	96 (10.3)
Bilateral lead placement	15 (1.6)
Lead details	
Oldest lead (y)	4.9 (1.9–8.1)
Combined lead age (y)	9.5 (3.6–16.9)
Transvenous leads	2.25 ± 0.76
Right atrial lead	804 (86)
RV pacing lead	405 (44)
RV high voltage lead (ICD)	576 (62)
Dual-coil ICD lead	458/576 (80)
Coronary sinus LV lead	237 (25)
Non-CS passive fixation lead(s)	171 (18)
Abandoned lead(s)	129 (14)
Epicardial lead(s)	20 (2)
RA leads	0.89 ± 0.39
RV pacing leads	0.46 ± 0.55
RV ICD leads	0.64 ± 0.52
CS LV leads	0.26 ± 0.45
Extraction indication	
Lead malfunction	490 (53)
Infection	273 (29)
Access and/or planned upgrade	136 (15)
Other	31 (3)

CRT = cardiac resynchronization therapy; CS = coronary sinus; ICD = implantable cardioverter-defibrillator; LV = left ventricular; RA = right atrial; RV = right ventricular.

### Extraction outcomes

**Figure 1:**
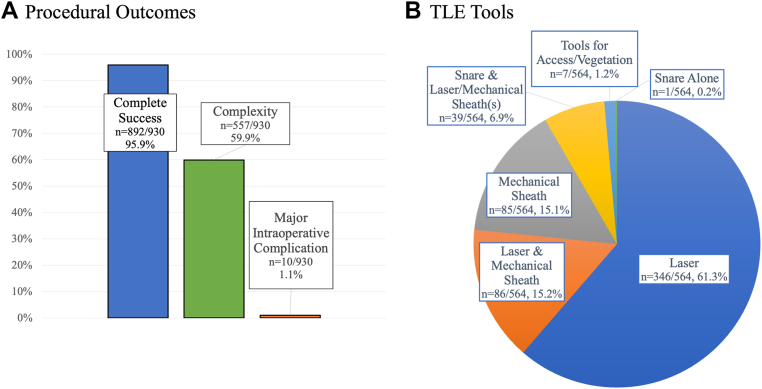
Procedural outcomes and TLE tools used. **A:** The frequencies of complete success, complexity, and major intraoperative complications. **B:** The breakdown of tools used for TLE. TLE = transvenous lead extraction.

The intent with TLE was full system removal in 571 TLEs (61.4%) (Table 3). Complete, clinical, or partial success occurred in 99.0% (Figure 1) (n = 921). Complete success was achieved in 892 TLEs (95.9%). The remainder included clinical success (n = 13; 1.4%), partial success (n = 16; 1.7%), or unsuccessful/abandoned (n = 9; 1.0%). There was an average of 1.71 ± 0.83 leads extracted per TLE. Complexity occurred with 59.9% of TLE (n = 557). Although tools were used in 60.6% of TLE (n = 564), in 7 patients (0.8%), complex tools were used predominantly to maintain access in the setting of occlusion or remove a lead mass (oldest lead age <2 years). In the remaining patients, complex tools were used for 59.9% of TLE (n = 574). This included the laser sheath (n = 346; 37.2%), laser and mechanical sheaths (n = 86; 9.2%), mechanical sheath (n = 85; 9.1%), snare and laser/mechanical (n = 39; 4.2%), and snare alone (n = 1; 0.1%). In the remaining TLE, lead removal was successful with traction only (366 TLEs; 39.3%). There were 39 total Bridge occlusion balloon (Philips, The Netherlands) deployments (4.2%), of which 37 were prophylactic only (4.0%).

**Table 3:** Extraction details and outcomes

Extraction details, outcomes, and complications	Overall (930 TLEs)
Extraction intent	
Full system extraction	571 (61.4)
Partial system extraction	359 (38.6)
Success	
Completely successful	892 (95.9)
Clinical success (but not complete owing to remnant <4 cm, including completely successful)	905 (97.3)
Partial success	16 (1.7)
Unsuccessful or abandoned	9 (1.0)
Leads extracted	
Total	1588
RA	549
RV pacing leads	383
RV ICD leads	523
CS LV leads	133
Lead extraction technique	
Traction only	366 (39.3)
Laser	346 (37.2)
Laser and mechanical	86 (9.2)
Mechanical	85 (9.1)
Laser/mechanical and femoral workstation	39 (4.2)
Femoral workstation only	1 (0.1)
Tools used predominantly for maintaining access/removal of a lead vegetation/mass	7 (0.8)
Extracted lead age (oldest)	
RA (y)	4.91 (1.84–9.20)
RV pacing (y)	3.72 (1.12–9.21)
RV ICD leads (y)	5.09 (2.82–7.52)
CS LV leads (y)	2.86 (0.67–5.44)
Complications	
Any complication (including with reimplant)	96 (10.3)
Major intraoperative complication	10 (1.1)
Major∗	15 (1.6)
Emergent cardiac surgery	9 (1.0)
Cardiac tamponade	6 (0.6)
SVC perforation without tamponade	5 (0.5)
TIA/stroke	3 (0.3)
Embolism with intervention†	1 (0.1)
Death during the procedure or owing to complication from the procedure	2 (0.2)
Minor∗	88 (9.5)
Pericardial effusion without intervention	3 (0.3)
Pneumothorax	2 (0.2)
Transfusion	7 (0.8)
DVT/PE	32 (3.4)
Hematoma requiring evacuation	19 (2.0)
Embolization of the lead component without intervention	2 (0.2)
Other complication‡	9 (1.0)

CS = coronary sinus; DVT = deep vein thrombosis; ICD = implantable cardioverter-defibrillator; LV = left ventricular; PE = pulmonary embolism; RA = right atrial; RV = right ventricular; SVC = superior vena cava; TIA = transient ischemic attack; TLE = transvenous lead extraction.

∗ Heart Rhythm Society 2017 criteria.

† Lead vegetation embolization with lung infarct resulting in hemoptysis requiring intubation for a few days prior to recovery.

‡ Other complications included. • 5 surgical site infections requiring antibiotics alone. • 1 surgical site infection requiring extraction. • 1 instance of postprocedure bleeding requiring transfusion (not caused by another complication above). • 1 right internal jugular fistula managed conservatively (from access at the temporary pacemaker site). • 1 vegetation embolization requiring reintubation after hemoptysis.

### Complications

There were 96 total complications (10.3%). This included 10 patients (1.1%) with a major intraoperative complication: 9 patients (1.0%) with emergent surgery (5 for cardiac perforation and 4 for superior vena cava [SVC] tear) and 1 intraoperative death (0.1%) on the table in a patient with *Staphylococcus aureus* bacteremia who developed cardiac tamponade and was not offered cardiac surgical backup before TLE owing to comorbidities. All patients with a major intraoperative complication had complex tools used during TLE (100%). There were 3 instances of a new/enlarged pericardial effusion (0.3%) during or after the procedure not requiring intervention. There were 2 deaths (0.2%) during the procedure or afterward attributable to a procedural complication (1 included the patient with tamponade who was deemed not a candidate for surgical repair preoperatively, and the other was because of complications from a massive stroke, which could have been caused by paradoxical embolism from lead fibrosis/material at the time of TLE). There were 2 Bridge balloon deployments (0.2%) owing to a complication, including the patient who died from cardiac tamponade and was deemed not a candidate for surgical repair before TLE and another patient with SVC tear with successful rescue and surgery.

### Factors associated with complexity

Univariate factors associated with complexity are presented in Table 4. Logistic regression analysis demonstrated that the following factors were independently associated with complexity: the number of ICD leads (*P* < .001), combined lead age (*P* < .001), the number of leads (*P* < .001), abandoned leads (*P* = .0045), patient age at the time of implantation of the oldest lead (*P* = .024), time since implantation of the oldest lead (*P* = .025), and patient age at time of TLE (*P* = .03).

**Table 4:** Factors associated with complexity (complications and/or need for complex tools)

Factors	Complexity∗ (n = 557)	Absence of complexity (n = 373)	*P* value	Logistic regression, OR (95% CI)	*P* value
Patient characteristics					
Age (y)	61.2 ± 16.0	65.5 ± 15.2	<.001†	1.27 (1.02–1.58)	.030†
Age at oldest lead implant (y)	52.6 ± 16.8	63.4 ± 15.5	<.001†	0.78 (0.63–0.97)	.024†
Men	383 (68.8)	253 (67.8)	.76		
Body mass index (kg/m^2^)	30.5 ± 6.9	30.5 ± 7.2	.99		
Left ventricular ejection fraction (%)	41.4 ± 15.6	42.0 ± 16.8	.61		
Hypertension	332 (59.6)	259 (69.4)	.002†	N/A	NS
Diabetes mellitus	170 (30.5)	122 (32.7)	.48		
Coronary artery disease	255 (45.8)	192 (51.5)	.089		
Previous sternotomy	154 (27.7)	110 (29.5)	.54		
Previous stroke	55 (9.9)	35 (9.4)	.80		
Chronic kidney disease	132 (23.7)	91 (24.4)	.81		
End-stage renal disease on hemodialysis	17 (3.0)	13 (3.5)	.71		
Atrial fibrillation	200 (35.9)	165 (44.2)	.011†	N/A	NS
Previous VT/VF or cardiac arrest	200 (35.9)	83 (22.3)	<.001†	N/A	NS
Device types					
Pacemaker	145 (26.0)	197 (52.8)	<.001†	N/A	NS
ICD	412 (74.0)	176 (47.2)	<.001†	N/A	NS
CRT	164 (29.4)	93 (24.9)	.13	N/A	NS
Left-sided device only	493 (88.5)	326 (87.4)	.61		
Right-sided device only	57 (10.2)	39 (10.5)	.91		
Bilateral lead placement	7 (1.3)	8 (2.1)	.29		
Lead details					
Time since implantation of the oldest lead (y)	8.59 ± 5.05	2.03 ± 2.46	<.001†	1.28 (1.03–1.59)	.025†
Combined lead age (y)	18.05 ± 12.63	4.11 ± 5.03	<.001†	1.32 (1.17–1.48)	<.001†
Number of transvenous leads	2.32 ± 0.81	2.14 ± 0.65	<.001†	0.42 (0.26–0.68)	<.001†
RA lead	482 (86.5)	322 (86.3)	.93		
RV pacing lead	191 (34.3)	214 (57.4)	<.001†	N/A	NS
RV high voltage lead (ICD)	410 (73.6)	166 (44.5)	<.001†	N/A	NS
Dual-coil ICD lead	349 of 410 (85.1)	109 of 166 (65.7)	<.001†	N/A	NS
CS LV lead	151 (27.1)	86 (23.1)	.16	N/A	NS
Passive fixation lead(s)–excluding CS	140 (25.1)	31 (8.3)	<.001†	N/A	NS
Abandoned lead(s)	105 (18.9)	24 (6.4)	<.001†	3.48 (1.48–8.18)	.0045†
Epicardial lead(s)	12 (2.2)	8 (2.1)	.99		
# of RA leads	0.90 ± 0.40	0.88 ± 0.37	.38		
# of RV pacing leads	0.38 ± 0.56	0.58 ± 0.51	<.001†	N/A	NS
# of RV ICD leads	0.76 ± 0.49	0.45 ± 0.50	<.001†	3.17 (1.99–5.04)	<.001†
# of CS LV leads	0.28 ± 0.46	0.24 ± 0.45	.21		
Extraction indication					
Lead malfunction	339 (60.9)	151 (40.50)	<.001†	N/A	NS
Infection	153 (27.5)	120 (32.2)	.12	N/A	NS
Access and/or planned upgrade	46 (8.3)	90 (24.1)	<.001†	N/A	NS
Other	19 (4.7)	12 (4.7)	.98		

CI = confidence interval; CRT = cardiac resynchronization therapy; CS = coronary sinus; ICD = implantable cardioverter-defibrillator; LV = left ventricular; N/A = not applicable; NS = nonsignificant; OR = odds ratio; VF = ventricular fibrillation; VT = ventricular tachycardia.

∗ Major intraoperative complication or need for complex tools.

† Statistically significant.

### Complexity with recently implanted leads

Unplanned complexity occurred for 2 of 128 pacemakers (1.6%) with the oldest lead being <2 years old and for 0 of 71 ICDs (0%) with the oldest lead being <1 year old (overall 2 of 203; 1.0%). There were no major intraoperative complications in this subset. 1 patient requiring complex tools was a 52-year-old man with a right-sided dual-chamber pacemaker (9-month-old leads) undergoing TLE owing to right atrial (RA) and right ventricular (RV) lead dysfunction. His RA lead was removed with traction, but the RV lead required a 14F laser for adhesions at the right subclavian-SVC junction (done in EP laboratory), which were likely in part related to a right subclavian occlusion. The second patient was a 37-year-old man undergoing TLE of a left-sided dual-chamber pacemaker with leads that were implanted 13 months earlier. He required an ICD upgrade and had a failed attempt at traction TLE at an outside hospital. Given this, the TLE was arranged in the hybrid room with cardiac surgical backup available. Both leads pulled back to the subclavian vein with the use of a locking stylet and traction but remained adherent under the clavicle. The RV lead was removed successfully with an 11F TightRail Sub-C followed by traction removal of the RA lead.

## Discussion

The key findings from this study included the following: (1) the number of ICD leads, combined lead age, number of leads, presence of abandoned leads, patient age at implantation, patient age at TLE, and time since implantation of the oldest lead were independent predictors of complexity with TLE; (2) there was a low frequency of major intraoperative complications (1.1%) with TLE; and (3) there was a very low likelihood of needing complex tools or a major intraoperative complication for recently placed leads (<2 years for PPM and <1 year for ICD). The strengths of this study include the inclusion of 9 operators, a large TLE volume over 12 years, and assessment of the validity of criteria for lower risk TLE done in the EP laboratory without cardiac surgical backup.

Although a few studies have looked at factors associated with the need for advanced TLE tools,^3^^,^^4^ complexity defined by second-line tools and TLE time,^5^ and major complications/mortality,^1^^,^^6^, ^7^, ^8^, ^9^, ^10^, ^11^ we used a combined definition for complexity including both advanced tools and complications with the aim of assisting future preprocedural planning and alignment of resources. Patient factors associated with an increased risk of complications or mortality include women,^6^, ^7^, ^8^^,^^10^^,^^11^ absence of previous cardiac surgery,^11^ low body mass index,^1^^,^^9^ renal insufficiency,^1^^,^^9^^,^^12^ diabetes,^9^^,^^11^ severe heart failure,^12^ infection,^9^ and age of younger than 30 years at the time of implantation.^10^ Younger age at the time of TLE has also been reported as being associated with an increased risk of needing advanced tools.^3^ Device/lead factors associated with an increased risk of complications or mortality include increased number of leads,^8^^,^^11^^,^^13^ longer lead dwelling time,^8^^,^^10^, ^11^, ^12^ and the presence of abandoned leads.^7^ These device/lead factors and the presence of passive fixation and/or ICD leads are also reported as associated with the need for more advanced tools and a more difficult procedure.^3^, ^4^, ^5^

Similarly, we found that age at TLE, age at implant, oldest lead age, combined lead age, the number of leads, abandoned leads, and the number of ICD leads were associated with complexity. There is no universal definition for more challenging TLEs, and others have used definitions based on fluoroscopy time, lead removal time, and need for second-line tools (beyond polypropylene sheaths).^3^, ^4^, ^5^ Our inclusion of both major intraoperative complications and the need for complex tools is important in considering whether a TLE can be successful with simpler tools alone without cardiac surgical backup.

Wazni et al^11^ reported a 0.3% risk of death owing to a major adverse event with TLE. We report a similarly low risk of 0.2%. Others report complete success and clinical success rates of 90.7–96.5% and 97.6%–97.7%, respectively.^1^^,^^14^^,^^15^ Similarly, we report complete success at 95.9% and clinical success at 97.3%. The reported rate of cardiac/vascular perforation requiring surgical intervention or resulting in death ranges from 1.0% to 2.7%.^1^^,^^11^^,^^16^ We report the need for a major intraoperative complication at 1.1% (including emergent cardiac surgery at 1.0%) and major complication as defined by HRS^2^ at 1.6%. The high success and low risk of major complications despite inclusion of some very high-risk TLEs highlight the programmatic success that can be achieved with appropriate protocols, close collaboration with CT surgery, and high clinical volume/experience.

Others have used predominantly lead age to stratify whether to proceed with TLE in the operating/hybrid room and whether or not to use cardiac surgical backup.^17^, ^18^, ^19^ Both Afzal et al^17^ and Kancharla et al^19^ report no major complications in low-risk TLE subsets. However, these studies do not specify how often complex tools were used in the low-risk subsets (in 2019, Kancharla et al^19^ reported a 46% frequency of laser utilization for low and intermediate risk combined). The use of complex tools increases the risk of major complications, and the low numbers in these studies are not sufficient for comfort in avoiding cardiac surgical backup. We report the likelihood of complexity (use of complex tools or major intraoperative complication) in a very-low-risk cohort defined by lead age with a pacemaker of <2 years and an ICD of <1 year. Similar to other studies, we report no major intraoperative complications in this subset. However, we do report a 1% risk of unplanned complexity in this cohort owing to need for complex tools. This has important implications on preprocedural planning and provides further confirmation that this is a very-low-risk TLE subset, which may not require the hybrid room and immediate cardiac surgical backup up front. Certainly, if there is escalation in risk and/or utilization of complex tools during TLE, either involving cardiac surgery for backup or abandoning the procedure and proceeding in the hybrid room with cardiac surgical backup would be warranted.

### Limitations

This is a retrospective single-center study. Thus, the findings may not be generalizable outside our institution. All operators were experienced at TLE, and most TLEs were performed initially with a superior approach. Furthermore, laser sheaths were used more frequently than mechanical sheaths. These results may not apply when the initial complex tool used is a femoral workstation or mechanical sheath. Although this study included lead explant procedures, this was only 8.3% of TLE and would not be expected to majorly skew the complications rate or findings. The inclusion of lead explants was important for the aim of this study. Only 3 TLEs were performed with conduction system pacing leads, and thus, these results are not generalizable to conduction system pacing leads. We report findings including years before the availability of the Bridge rescue balloon. Although this balloon was used in later years of this study, the risk of morbidity and mortality may be elevated in this study compared with current practices owing to the current availability of this rescue option (and frequent prophylactic usage). Cardiac perfusionists were in the room, and our cardiac surgeons were available in a timely manner either in the room or in close proximity during TLE in the hybrid room. The prompt availability of the cardiac surgical team likely contributed to the low mortality rate reported, and this type of collaboration may not be generalizable to all institutions.

## Conclusion

The frequency of major intraoperative complications was low (1.2%), and all occurred in patients in whom complex tools were used for TLE. Factors associated with complexity included the presence of an ICD, abandoned leads, patient age, and the number/age of leads. The risk of unplanned complexity (major intraoperative complication or need for complex tools) is very low for pacemaker leads <2 years and ICDs <1 year since implantation. This subset can be considered for TLE in the EP laboratory without immediate cardiac surgical backup.

### Clinical implications

TLE may be reasonable in the EP laboratory without immediate cardiac surgical backup in a subset of patients with the oldest pacemaker leads <2 years and ICD leads <1 year since implantation. There is a higher risk of complexity (need for complex tools or major intraoperative complication) with a higher number of ICD leads, combined lead age, number of leads, presence of abandoned leads, patient age at TLE, and time since implantation of the oldest lead. Younger patients at implantation also have a higher risk of complexity.

## Disclosures

S.H.S.: Abbott (honoraria for educational conference), Biosense Webster (consulting), Boston Scientific (honoraria for educational conference), and Medtronic (honoraria for educational conference and consulting). No other authors have conflicts of interest to disclose.
